# 637. Stable bloodstream infection rates despite rising colonization: Insights from a hospital system using in-house PCR screening for *Candida auris*

**DOI:** 10.1093/ofid/ofae631.202

**Published:** 2025-01-29

**Authors:** Rossana M Rosa, Gemma Rosello, Kelley Manzanillo, Octavio Martinez, Lilian M Abbo

**Affiliations:** Jackson Health System , Fredericton, New Brunswick, Canada; Jackson Health System, Miami, Florida; Jackson Health System, Miami, Florida; Jackson Health System/University of Miami, Miami, FL; University of Miami Miller School of Medicine, Jackson Health System, Aventura, FL

## Abstract

**Background:**

*Candida auris* (*C. auris*) is spreading in the United States at a rapid pace. At our facilities, risk-based admission screening for colonization with *C. auris* using PCR has been a cost-effective tool for guiding infection prevention (IP) practices. We describe the observed trends in incidence of detection of colonization present on admission and hospital-onset cultures at our facilities since deploying in-house PCR testing.

**Figure 1:**
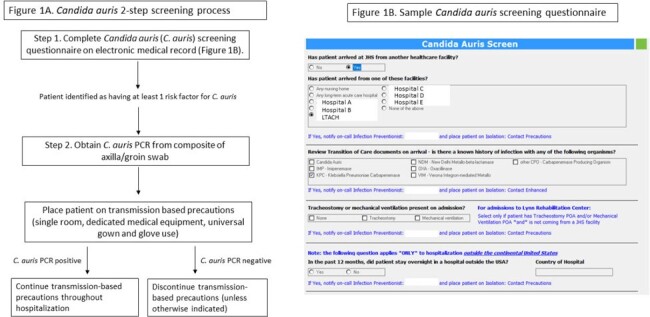
A. Candida auris 2-step screening process B: Sample Candida auris screening questionnaire

**Methods:**

Study conducted at an integrated healthcare system in Miami, Florida, consisting of a large tertiary care center, three community hospitals and one inpatient rehabilitation hospital. The study period went from August 2021 to December 2023. In August 2021 we implemented admission screening for *C. auris* with PCR performed in-house. Screening criteria, testing and isolation protocols are presented in Figure 1A-B. Patients testing negative on admission were not routinely re-tested. Joinpoint regression was used to estimate changes in the incidence rates of *C. auris* newly present on admission surveillance (CA-POA), hospital-onset (HO) clinical cultures from any source and bloodstream infections (BSI). Definitions used are presented in Table 1.

**Figure:**
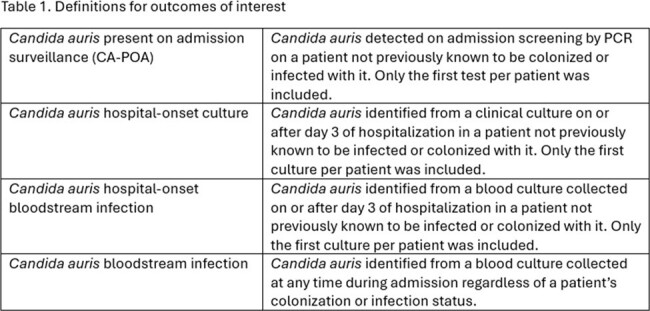

**Results:**

We identified 292 patients with CA-POA, for a crude rate of 9.1 per 10,000 patient-admissions. Between August 2021 and June 2023, the monthly percent change in the incidence rate of CA-POA significantly increased by 4.9% (95% CI 2.6-7.2; p< 0.01) and decreased by 17.4% (95% CI -30.8 to -1.5; p=0.03) between July 2023 and December 2023. There were 124 patients with HO clinical cultures, including 72 hospital-onset BSIs, for a crude BSI rate of 0.59 per 10,000 patient-days. There were no statistically significant changes in the trends of incidence of these infections during the study period. Incidence rates for the different outcomes of interest are displayed in Figure 2A-2D.

Incidence rates for outcomes of interest

**Figure:**
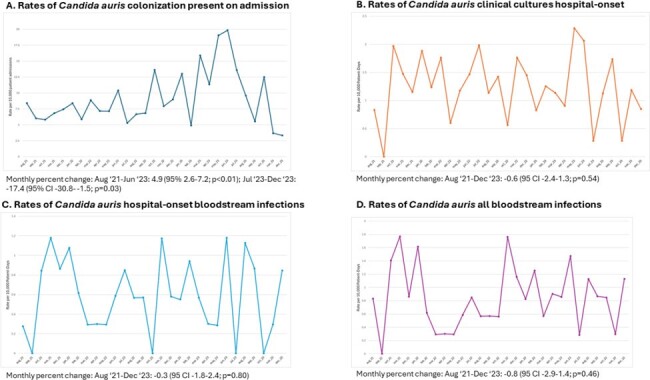

**Conclusion:**

We found that despite rising rates of patients newly identified as already colonized with *C. auris* upon admission, the rates of HO cultures and most importantly of bloodstream infections have remained stable at our facilities. Admission screening using in-house PCR can lead to a more precise deployment of IP resources, mitigation of horizontal transmission and raising public health awareness on spread occurring outside hospitals.

**Disclosures:**

**All Authors**: No reported disclosures

